# Streptothricin acetyl transferase 2 (*Sat2*): A dominant selection marker for *Caenorhabditis elegans* genome editing

**DOI:** 10.1371/journal.pone.0197128

**Published:** 2018-05-09

**Authors:** Hiroyuki Obinata, Asako Sugimoto, Shinsuke Niwa

**Affiliations:** 1 Division of Developmental Dynamics, Graduate School of Life Science Tohoku University, Aoba-ku, Sendai, Japan; 2 Frontier Research Institute for Interdisciplinary Sciences (FRIS), Tohoku University, Aoba-ku, Sendai, Japan; INSERM U869, FRANCE

## Abstract

Studies on *Caenorhabditis elegans* would benefit from the introduction of new selectable markers to allow more complex types of experiments to be conducted with this model animal. We established a new antibiotic selection marker for *C*. *elegans* transformation based on nourseothricin (NTC) and its resistance-encoding gene, streptothricin-acetyl transferase 2 (*Sat2*). NTC was able to efficiently prevent worm development at very low concentrations, and the worms expressing *Sat2* were able to survive on the selection plates without any developmental defects. Using CRISPR/Cas9 and NTC selection, we were able to easily insert a 13-kb expression cassette into a defined locus in *C*. *elegans*. The structure and spectrum of NTC differs from other antibiotics like hygromycin B and geneticin, making it possible to use NTC alongside them. Indeed, we confirmed NTC-*sat2* selection could work with the hygromycin B selection system simultaneously. Thus, the new NTC–*Sat2* system can act as a useful dominant marker for gene transfer and genome editing in *C*. *elegans*.

## Introduction

The nematode *Caenorhabditis elegans* has been used to study a broad spectrum of biological problems in many fields. One of the main reasons for the success of *C*. *elegans* as a model organism is the ease by which it undergoes gene transduction. Transgene expression in *C*. *elegans* is accomplished by microinjection of DNA into the gonads [1]. Several selectable genetic markers have been used [2]. Traditionally, transformed animals are identified by rescuing non-lethal mutations [3, 4] via the expression of a dominant phenotype marker [1] or a fluorescent marker [5, 6]. *C*. *elegans* can inherit DNA molecules injected into it as extrachromosomal arrays [7]. However, because extrachromosomal arrays tend to be inherited in only a subset of the progeny worms, it is difficult to grow large populations of transgenic strains. Moreover, to maintain the extrachromosomal array-carrying strains, researchers need to pick up individual worms one by one using readily distinguishable markers [1, 7].

Antibiotics and their corresponding resistance genes are now used widely as markers for gene transfer in many organisms such as bacteria and yeast, and in mammalian cells also. One advantage of these markers is that the transgene can be introduced into any genetic background. Antibiotic selection systems have been applied to worms as well, and some pairs of antibiotics and their resistance genes that work in nematodes have been reported (e.g. puromycin [8], geneticin (G418) [9], hygromycin B [10, 11] and blasticidin S [12]). Nevertheless, from the perspectives of cost, ease of use and efficiency, designing more transgenic markers would be very useful.

Nourseothricin (NTC), a member of the streptothricin class of aminoglycoside antibiotics, inhibits cellular protein synthesis by inducing miscoding [13]. Prokaryotic and eukaryote cells alike are efficiently killed by NTC. NTC and its associated resistance genes have already been used in bacteria, yeast, plants and mammalian cells as selection markers [14–16]. One of the NTC resistance genes, streptothricin acetyl transferase 2 (*Sat*2), was originally derived from a bacterial transposon, and confers streptothricin resistance to host strains [17]. *Sat2* is a 174-amino-acid protein that inactivates the streptothricin class of aminoglycoside antibiotics, including NTC, by its N-acetyltransferase activity. As the structure and spectrum of NTC are totally different from other aminoglycoside antibiotics such as G418 or hygromycin B, *Sat2* would not inactivate these antibiotics. Thus, *Sat2* and the resistance genes for such drugs can be used simultaneously as selection markers in human cell cultures [16].

Genome editing by CRISPR/Cas9 has been widely used in the field of *C*. *elegans* genetics. A lot of genome editing techniques such as single copy insertion and homologous recombination rely on antibiotics selection [18, 19]. Therefore, the availability of additional selection markers that can be compatible with genome editing will enable more complex and innovative experimental designs to be considered by researchers.

In this study, we report that NTC and its resistance gene *Sat2* can act together as a dominant selection marker for *C*. *elegans* transgenesis. In wild-type worms, growth and development were efficiently inhibited in the presence of NTC, whereas the *Sat2*-expressing worms were able to develop normally and reproduce on the selection medium. In terms of the price per experiment, the cost of this system is comparable to other antibiotic selection systems, making it a good choice for gene transfer in *C*. *elegans*. Notably, we have shown in our experiments that NTC selection is compatible with CRISPR/Cas9-mediated single-copy insertion, as revealed by cell-specific expression following insertion of an expression cassette of ~ 13-kb into a defined genomic locus. Moreover, we confirmed that our NTC-*Sat2* selection system was also able to work with the hygromycin B selection system at the same time.

## Materials and methods

### Worm strains and maintenance

Wild-type *C*. *elegans* (N2 Bristol strain), obtained from the *C*. *elegans* Genetic Center (CGC) (https://cgc.umn.edu/), was grown in standard nematode growth medium (NGM) [20]. We used 60-mm plates containing 12 mL of NGM with 100 μl of OP50 feeder cultured on the NGM. All strains were maintained at 20°C. In addition to N2, the following strains were used in this study: OTL90: jpnSi5[[*Punc-4c*::*Cre*, *Prps-0*::*Sat2*, *Pmig-13*::*loxP*::*STOP*::*loxP*::*sam-4*::*gfp*] V], OTL94: jpnSi7 [[*Punc-4c*::*Cre*, *Prps-0*::*Sat2*, *Pmig-13*::*loxP*::*STOP*::*loxP*::*tomm-20*::*gfp*] V], OTL80: jpnSi1 [[*Punc-4c*::*Cre*, *Prps-0*::*HygR*, *Pmig-13*::*loxP*::*STOP*::*loxP*::*tomm-20*::*gfp*] II], OTL95: jpnEx65 [*Pmyo-2*::*mCherry*, *Pmyo-3*::*mCherry*, *Prab-3*::*mCherry*, *Prps-27*::*Sat2*], and OTL96: jpnEx66 [*Pmyo-2*::*mCherry*, *Pmyo-3*::*mCherry*, *Prab-3*::*mCherry*, *Prps-27*::*Sat2*].

### Plasmid construction

PCR was performed using KOD-Plus-Neo (TOYOBO, Tokyo, Japan) following the manufacturer’s protocol. To generate *Prps-27*::*Sat2* vector (pSN444, addgene ID 108331) and *Prps-27*::*Nat* vector, the codon-optimized NTC resistance genes *Sat2* (GenBank: AKT95189.1) and nourseothricin acetyl transferase (*Nat*) (GenBank: AAC60439.1) were synthesized by Gene Art (Invitrogen, Carlsbad, CA, USA). The *rps-27* promoter was obtained from pCFJ910 (a gift from Erik Jorgensen, Addgene#44481). The fragments were inserted into the ΔpSM vector (a generous gift from Dr. Kang Shen, Stanford University) using *Asc*I/*Kpn*I and *Sph*I/*Asc*I restriction enzyme sites.

To construct the pOB4_*tomm-20* repair template vector, we first generated the pOB4 parental vector. To insert the expression cassette into the genome, we designed a Cas9 target site on chromosome II (position 8,420,204), the locus of which has been reported as the insertion site of *ttTi5605* for mos1-mediated single-copy insertion (MosSCI) [21]. The Cas9 target site was chosen using the online design tool available at CRISPRdirect (http://crispr.dbcls.jp/) [22]. The target sequence is 5'-GATATCAGTCTGTTTCGTAACGG-3'. The underline shows the protospacer adjacent motif (PAM) sequence. As homology arms, 700–900 base pair (bp) DNA fragments were amplified by polymerase chain reaction (PCR) from the N2 genome using 5'-tcgacgcaagcttctccttc-3' (sense primer) and 5'-cgaaacagactgatatcgaaac-3' (antisense primer) for the left arm, and 5'-taacggtcttctgtataactac-3' (sense primer) and 5'-tggaggccttgttgtcgatc-3' (antisense primer) for the right arm. *Punc-4c* and *Pmig-13*::*loxP*::*STOP*::*loxP* have been described previously [23]. *Prps-0*::*HygR*::*unc-54* 3′UTR was amplified from pDD282 (a gift from Bob Goldstein, Addgene#66823 [18]). *Punc-4c*::*Cre*::*tbb-2* 3′UTR and *Pmig-13*::*loxP*::*STOP*::*loxP*::*gfp*::*rab-3* 3′UTR vectors were constructed and then assembled with the homology arms, *Prps-0*::*HygR*::*unc-54* 3’UTR and *Sac*I-*Kpn*I digested pBlueScript II KS(-) by the Gibson assembly method [24]. The *tomm-20* fragment was PCR-amplified from worm cDNA and ligated to the *Sal*I digested parental pOB4 plasmid using the Gibson assembly method. To construct the pOB5_*sam-4* and pOB5_*tomm-20* repair template vectors, we first generated the pOB5 parental vector by the same method described above for pOB4. The Cas9 target locus for pOB5 was designed for chromosome V (position 8,643,066), the locus of which has been reported as the insertion site of *oxTi365* for universal MosSCI [25]. The Cas9 target site was also chosen using CRISPRdirect. The target sequence is 5'-CGGTCGCGGTACTCCAAATAGG-3'. The underline shows the PAM sequence. As homology arms, 700–900 bp DNA fragments were PCR-amplified from the WRM0613cH01 fosmid using 5'- agcgttgatgattggagggg-3' (sense primer) and 5'- cggtcgcggtactccaaat-3' (antisense primer) for the left arm, and 5'- aggcaggatattctccatttctga-3' (sense primer) and 5'- caaacaccacgcacattccc-3' (antisense primer) for the right arm. The *tomm-20* fragment and *sam-4* cDNA [26] were PCR-amplified and ligated to the *Sal*I digested pOB5 construct using the Gibson assembly method.

pTK73_pOB4 and pTK73_pOB5 were used to express single guide RNA (sgRNA) for Cas9-mediated insertion by pOB4 and pOB5. To construct the pTK73_pOB4 and pTK73_pOB5 sgRNA-expressing vectors, we first generated the sgRNA(F+E)-expressing parental vector, pTK73 [27] [28]. The sgRNA(F+E) scaffold was introduced into pRB1017 (a gift from Andrew Fire, addgene#59936 [29]). The sgRNA(F+E) scaffold sequence was obtained by PCR and then ligated to *Xho*I-*Nhe*I digested pRB1017. The target sequence for Cas9-mediated insertion are 5'-GATATCAGTCTGTTTCGTAACGG-3' for pOB4 (chromosome II) and 5'-CGGTCGCGGTACTCCAAATAGG-3' for pOB5 (chromosome V) as described above. Underlines indicate PAM sequences. Therefore, the primers used to insert the Cas9 target sequence into pTK73_pOB4 comprised 5'-TCTTGatatcagtctgtttcgtaa-3' and 5'-AAACttacgaaacagactgatatC-3', whereas those for pTK73_pOB5 comprised 5'-TCTTGcggtcgcggtactccaaat-3' and 5'-AAACatttggagtaccgcgaccgC-3', all of which were commercially synthesized by Eurofin Genetics Japan (Tokyo, Japan). The primers were annealed and ligated to *Bsa*I-digested pTK73 as described previously [29].

### NTC survival assays

NTC was purchased from Jena Bioscience (Germany). We reconstituted the NTC powder in double-distilled water (DDW) to each concentration and stored the aliquots at −30°C. For the antibiotic selection experiments, the NTC solutions were added directly to the growth plates at final concentrations ranging from 0.02 mg/mL to 2.00 mg/mL (NTC weight/ NGM volume). The plates were air dried with their lids off. To determine the optimal concentration of NTC, we placed more than ten synchronized L1 worms or ten gravid young adult worms onto each selection plate concentration. The three plates prepared for each concentration were monitored for up to four days for the larvae or seven days for the adults.

### NTC selection assays

To generate the *Sat2*-carrying extrachromosomal array strains, we injected 20 ng/μL of *Prps-27*::*Sat2* vector (pSN444) with 60 ng/μl of pBlueScript II KS(-) and three *mCherry* markers [2.5 ng/μl of pCFJ90 (*Pmyo-2*::*mCherry*), 5.0 ng/μl of pCFJ104 (*Pmyo-3*::*mCherry*) and 10 ng/μl of pGH8 (*Pmyo-3*::*mCherry*), all of which were gifts from Erik Jorgensen Addgene #19327, #19328 and #193595 [6]], into the N2 worms. At the F1 generation, we picked up mCherry-positive worms under an SZX16 stereoscopic fluorescence microscope (Olympus, Tokyo, Japan). Lines stably expressing mCherry after the F2 generation were selected. For the enrichment experiments, we placed ten mCherry-positive adult worms onto NGM plates with or without 0.04 mg/ml of NTC. Two independent strains were tested on two plates each. After three days of incubation, the worms were transferred to new selection plates or non-selection plates by removing 25-mm^2^ of the gel from each plate. This procedure was repeated four times. After the final transfer, the effect of *Sat2* expression was evaluated by calculating the ratio of mCherry-positive worms to the total number of worms.

### NTC-*Sat2*-based selection combined with CRISPR/Cas9

CRISPR/Cas9-induced genome editing was performed as described previously [19] with one modification. Repair template vector (10 ng/μL; pOB5_*sam-4*), 50 ng/μL of sgRNA vector (pTK73_pOB5) and 50 ng/μL of pDD162 (*Peft-3*::*Cas9*, a gift from Bob Goldstein, Addgene #47549 [18]), were co-injected into the N2 worms with three *mCherry* markers [pCFJ90 (*Pmyo-2*::*mCherry*), pCFJ104 (*Pmyo-3*::*mCherry*) and pGH8 (*Pmyo-3*::*mCherry*)]. After injection, the P0 parents were placed on normal NGM plates with OP50 feeder and allowed to lay eggs. One day later, 500 μl of NTC solution was added directly to the plates at its optimal concentration (final 0.04 mg/mL, NTC weight/NGM volume) and the plates were allowed to dry. After 7–10 days of incubation at 20°C, either the extrachromosomal array-carrying worms (mCherry-positive) or the single-copy inserted worms (mCherry-negative) survived on the selection plates. Next, we picked up L4 or adult worms that looked healthy but lacked mCherry fluorescence (i.e. lacked the extrachromosomal array markers) under the stereoscopic fluorescence microscope and singled them out onto new nonselective NGM plates. Integration was checked by worm PCR, followed by observation of SAM-4::GFP using microscopy. The Tomm-20::GFP strain was obtained using the same stream.

### Body bending assays

The thrashing rate assay was performed as described by Mikiro and Masaaki (2012) [30]. Young-adult worms (containing less than 7 eggs) were transferred to an NGM plate (φ = 35 mm) filled with 1mL of M9 buffer (3 g KH2PO4, 6 g Na2HPO4, 5 g NaCl, 1 mL 1 M MgSO4 and H2O to 1 liter). To become accustomed to the environment, the worms were left for 1 min in the plates at room temperature. After that, we recorded worm bending movies for 1 min and counted the number of thrashes. Twenty worms per strain were used in each bending assay.

### Double selection by NCT and hygromycin B

Hygromycin B Gold (solution) was purchased from nacalai tesque (Kyoto, Japan). Hygromycin B solution was diluted with DDW to a final concentration of 0.08 mg/mL (hygromycin B weight/ NGM volume) just before addition to the plates. Both NTC solution and hygromycin B solution were added directly to the NGM plates already seeded with OP50. The OTL80 strain with hygromycin B resistance (integrated *HygR* with *tomm-20*::*gfp* in chromosome II) was used. Male OTL80 worms were crossed with OTL90 (integrated *Sat2* with *sam-4*::*gfp* in chromosome V) hermaphrodite worms and F1 worms were obtained. We put 10 young adult F1 worms onto each selection plate containing NTC and/or hygromycin B. After 3 days incubation, we checked the SAM-4::GFP and TOMM-20::GFP signals from the F2 worms and calculated the ratio for each worm.

### Microscopy

The worm images on the plates were acquired using an SZX16 stereoscopic fluorescence microscope (Olympus) equipped with a CoolSNAP HQ cooled-CCD camera (Photometrics, Tucson, AZ, USA). Before image acquisition, the plates were cooled at −30°C for 1 min to prevent worm movements. For the fluorescence imaging of the *sam-4*::*gfp* integrated worms, we used an ORCA-R2 digital CCD camera (Hamamatsu Photonics, Hamamatsu, Japan) on an IX71 microscope (Olympus) with an UPlanSApo 60x/ NA1.3 silicone oil objective lens (Olympus) and a CSU-X1 spinning disc confocal system (Yokogawa Electric Corporation, Tokyo, Japan). Worms were mounted onto 3% agar pads with 2.5mM levamisol in M9 buffer, as described previously [31]. MetaMorph software (Molecular Devices, Sunnyvale, CA, USA) was used to control both microscopes. Fluorescent images of the worms were taken with 40 Z-slices and 1 μm intervals. ImageJ (NIH, https://imagej.nih.gov/ij/index.html) was used to process the images acquired.

### Worm PCR

To confirm that the genome editing occurred successfully at the proper locus, we performed worm PCR using the following primer pairs: P1: 5'-cgtcggcatcacaaggaatg-3', P2: 5'-tcatcacttgtcgcgtcaac-3', P3: 5'-agttatcgctagcagggcac-3' and P4: 5'-cgtcggcatcacaaggaatg-3'.

Ten adult worms were picked into 10 μl of lysis solution (10mM Tris-HCl, pH 8.2; 50mM KCl, 2.5mM MgCl_2_, 0.45% NP40, 0.45%Tween 20, 0.01% gelatin, and 0.5 mg/mL proteinase K) to extract their genomic DNAs. After one h digestion at 60 °C, the solutions were heated to 90°C for 15 min to inactivate the proteinase K. These solutions were then used directly as the PCR templates. Takara Ex Taq was used as described in the manufacturer’s protocol for the PCRs (Takara, Tokyo, Japan).

### Statistics

Statistical analyses were performed by using GraphPad PRISM 7 (GraphPad, La Jolla, CA, USA) on Mac OSX (Apple Inc., Cupertino, CA, USA). Statistical methods and sample numbers are clearly described in each graph or figure legends.

## Results

### The NTC-resistance gene *Sat2* works as a selection marker in *C*. *elegans*

**Fig 1:**
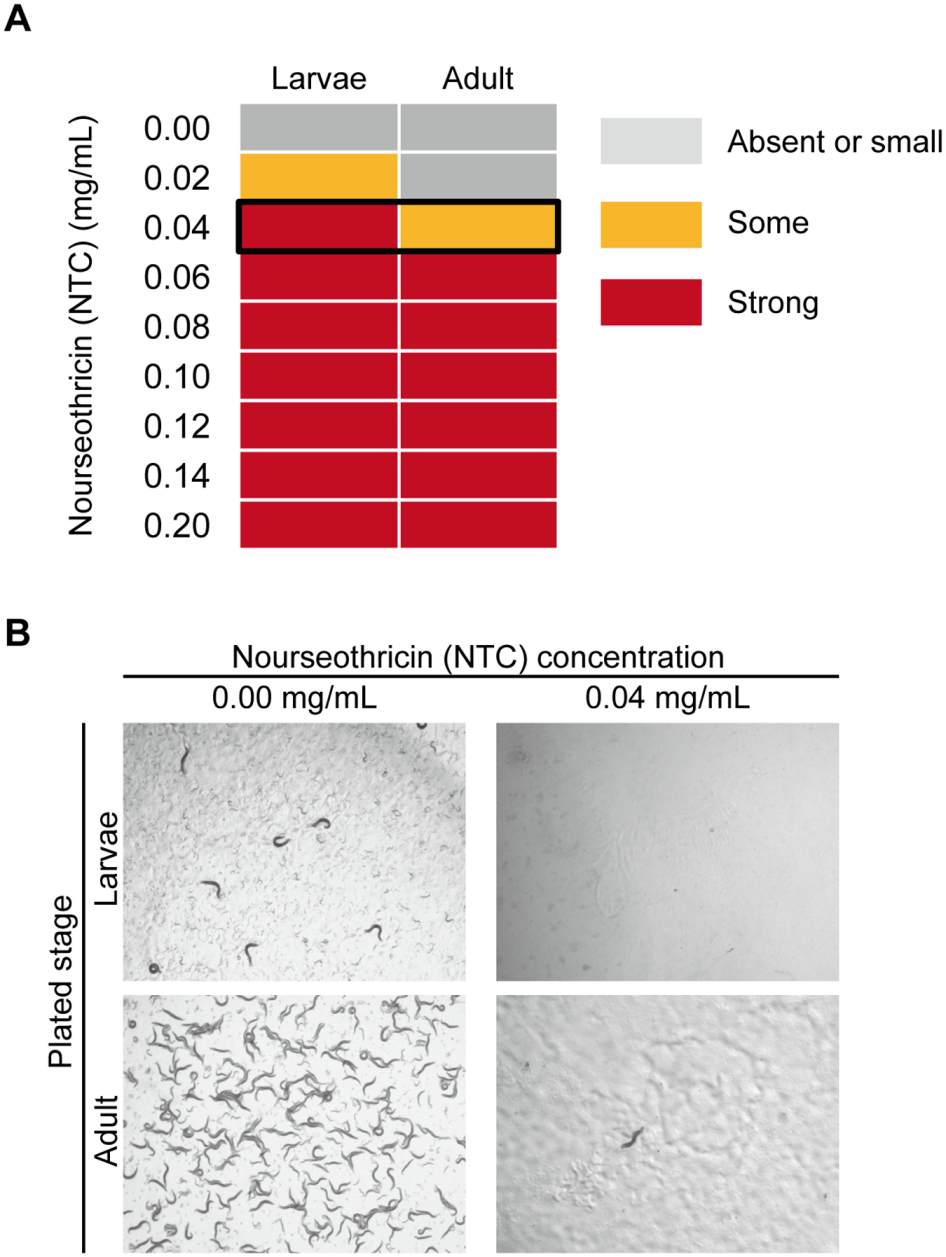
Viability assay in wild-type *C*. *elegans* treated with nourseothricin. (A) The effectiveness of nourseothricin on wild-type worms was divided into 3 classes. Absent or small: The effect was absent or slightly small during larvae development or in adult worm maintenance. Some: some larvae had arrested growth at the L1/ L2 stage, others developed to adulthood, but the adult worms died earlier than the controls but laid sufficient numbers of eggs. Strong: larval development stopped completely at the L1/L2 stage, or almost all the adults died a few days after adding the NTC solutions. The black frame indicates the optimal concentration of NTC. (B) Micrographs showing larvae and adult plates at the optimal concentration (0.04 mg/mL). Both pictures were taken four days after worm placement.

In the search for alternative antibiotics and antibiotic resistance genes that can be applied to *C*. *elegans* genetics, we found that NTC can kill wild-type worms efficiently (Fig 1). The young larvae were found to be more sensitive to NTC than the adult worms, perhaps because they need more rapid protein synthesis for development. In *C*. *elegans* transformations, DNA is injected into the gonads of the parental worms and the next generation is scored [1]. Thus, to obtain as many transgenic progenies as possible, the adult worms need to live until they stop laying eggs, while larvae are unable to develop on the selection plate. At 0.04 mg/mL (NTC weight/NGM volume), growth of wild-type larvae was completely stopped, while adult worms could live for several days. Thus, we defined that 0.04 mg/mL is the optimal quantity of NTC to use in the experiments (Fig 1A and 1B).

**Fig 2:**
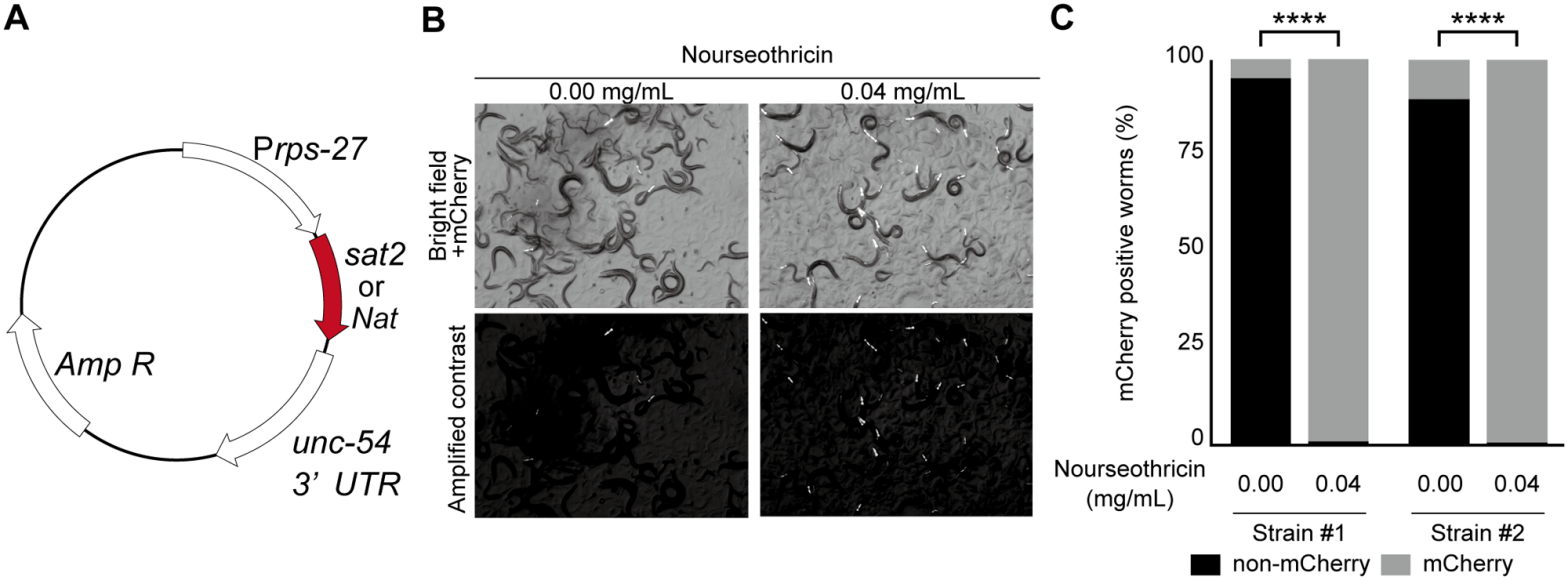
*Sat2*-carrying worms can develop in the optimal concentration of nourseothricin. (A) Schematic of the resistance-marker vector. The vector contains the NTC resistance genes, *Sat2* or *Nat*, under the control of the universal ribosomal promoter, *rps-27*. (B and C) Ten mCherry-positive P0 worms were placed onto selective or nonselective plates and, after three days of incubation, a 25 mm^2^ slice was excised from each gel and transferred to new selective or nonselective plates, respectively. Transfers were repeated 4-times and the plates were scored three days after the final transfer. (B) Micrographs showing worms carrying *Prps-27*::*Sat2*, *Pmyo-2*::*mCherry*, *Pmyo-3*::*mCherry* and *Prab-3*::*mCherry* as an extrachromosomal array. Upper panels show the original bright images of the worms and the mCherry signals. Lower panels, the contrast images to observe mCherry signal. (C) The plot shows the ratios of mCherry-positive to mCherry-negative worms. The results for two independent strains are shown. ****, p < 0.0001, Fisher’s exact test, n = 224 (line #1, 0.00 mg/ml), 226 (line #1, 0.04 mg/ml), 215 (line #2, 0.00 mg/ml) and 273 (line #2, 0.04 mg/ml) worms. Note that most worms have extrachromosomal arrays even after the 4-times transfer when cultured on the NTC-containing plates. In contrast, the extrachromosomal arrays were largely lost from the worms on the non-selection plates.

We next tested two NTC resistance genes, *Sat2* and *Nat*, as potential selection markers for *C*. *elegans* transformation [14, 16]. In order to express these genes in *C*. *elegans*, *Sat2* and *Nat* were codon optimized using a *C*. *elegans* codon adaptor [32] (https://worm.mpi-cbg.de/codons/cgi-bin/optimize.py) and then synthesized. As seen with other drug-resistance genes, *Sat2* and *Nat* are each expressed under the control of a universal promoter for the ribosomal protein encoding gene, *rps-27* [8] [9] (Fig 2A). We injected *Prps-27*::*Sat2* or *Prps-27*::*Nat* vectors, which each contain three visible *mCherry* fluorescence markers [pCFJ90 (*Pmyo-2*::*mCherry*), pCFJ104 (*Pmyo-3*::*mCherry*) and pGH8 (*Pmyo-3*::*mCherry*)], into N2 worms and obtained transgenic strains that contained the extrachromosomal arrays. Using mCherry as a visible marker, we placed ten extrachromosomal array-positive worms per strain onto selection plates. Subsequently, the *Sat2*-expressing worms but not the *Nat*-expressing worms survived on NGM agar containing NTC. As we were unsure of the exact reason why *Nat* was ineffective in the worms, we focused our efforts on *Sat2* thereafter.

Extrachromosomal arrays become lost from the germ line at various frequencies. Thus, if extrachromosomal array-containing worms are not selected, the arrays are often lost after several generations. Therefore, to test whether *Sat2* is useful for maintaining the extrachromosomal arrays, we performed the following experiment. The extrachromosomal array-positive worms were placed onto selective or non-selective plates. When the plates became starved, the worms were transferred to new selection or non-selection plates by cutting the gel containing them off by 25 mm^2^. We repeated this procedure four times. After the final transfer, the maintenance of the extrachromosomal arrays was evaluated by calculating the ratio of mCherry-positive worms to total worm numbers (Fig 2B and 2C). When the worms were cultured on plates lacking NTC, the average ratio of the transgenic worms was 7.5% of the total worms. However, when the worms were cultured on the selection plates, almost all the worms had mCherry signals (Fig 2C), indicating that NTC and *Sat2* helped to enrich the population of the extrachromosomal array strains.

### NTC selection is useful for Cas9-mediated single-copy insertion

**Fig 3:**
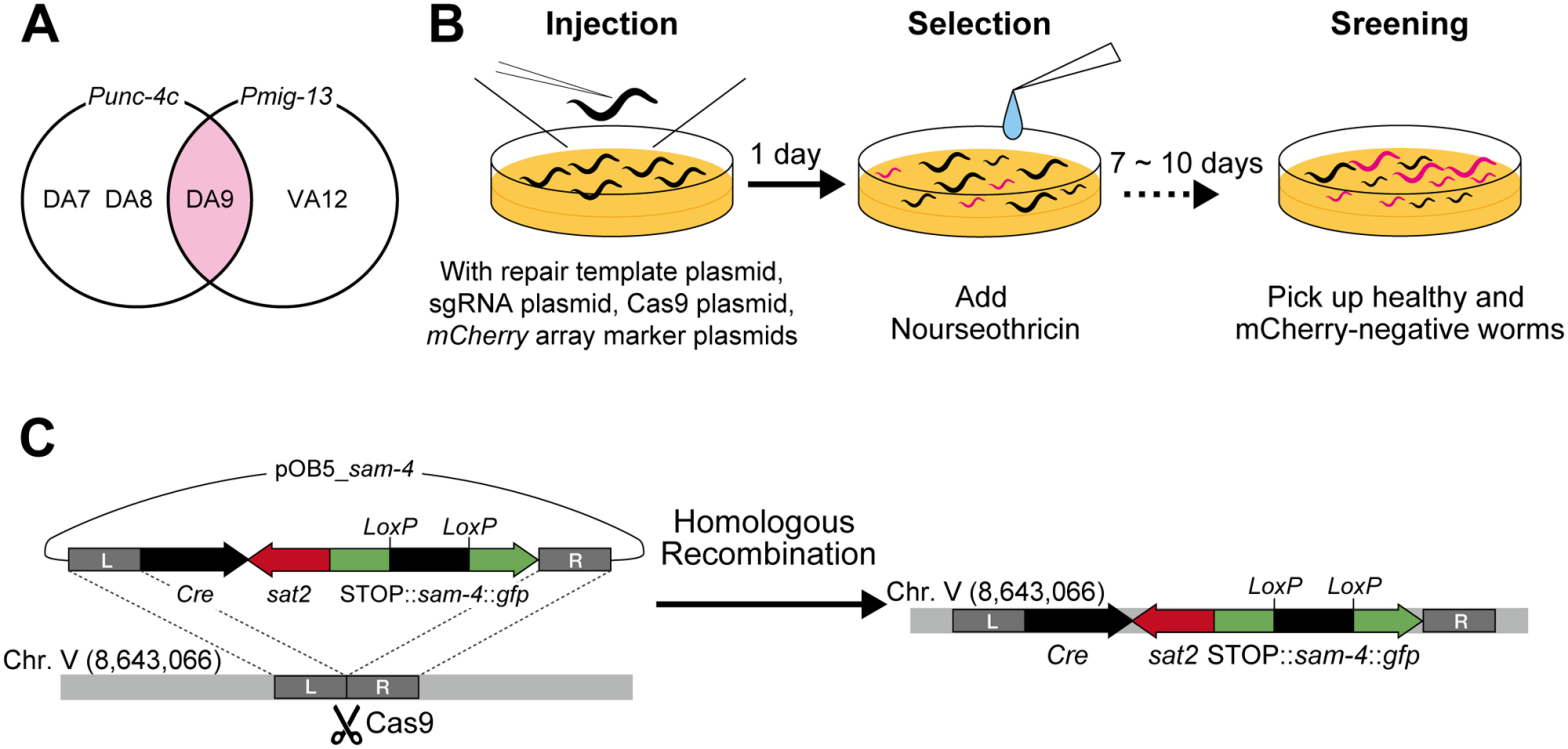
Nourseothricin selection process for obtaining single-copy insertion using CRISPR/Cas9. (A) Venn diagram showing the *unc-4c* promoter- (*Punc-4c*) and *mig-13* promoter- (*Pmig-13*) expressing neurons. (B) Schematic of the protocol used to identify worms carrying the single-copy insert. The repair template vector, sgRNA vector, *Cas9* vector, and three *mCherry* vectors are injected to N2 worms. Next day, the NTC solutions are added and the worms are allowed to grow for 7–10 days. After incubation, the healthy-looking L4/adult worms that have no mCherry signal (extrachromosomal array marker) are picked up. (C) Schematic showing the experimental design used for inserting the DA9-specific expression cassette into the defined locus. The target site for Cas9 was designed on chromosome V. Using the homology arms around the target locus, the expression cassette was inserted by homologous recombination. The resultant worms carry Cre recombinase, NTC resistance and the expression cassette, but do not carry mCherry (extrachromosomal array markers).

**Fig 4:**
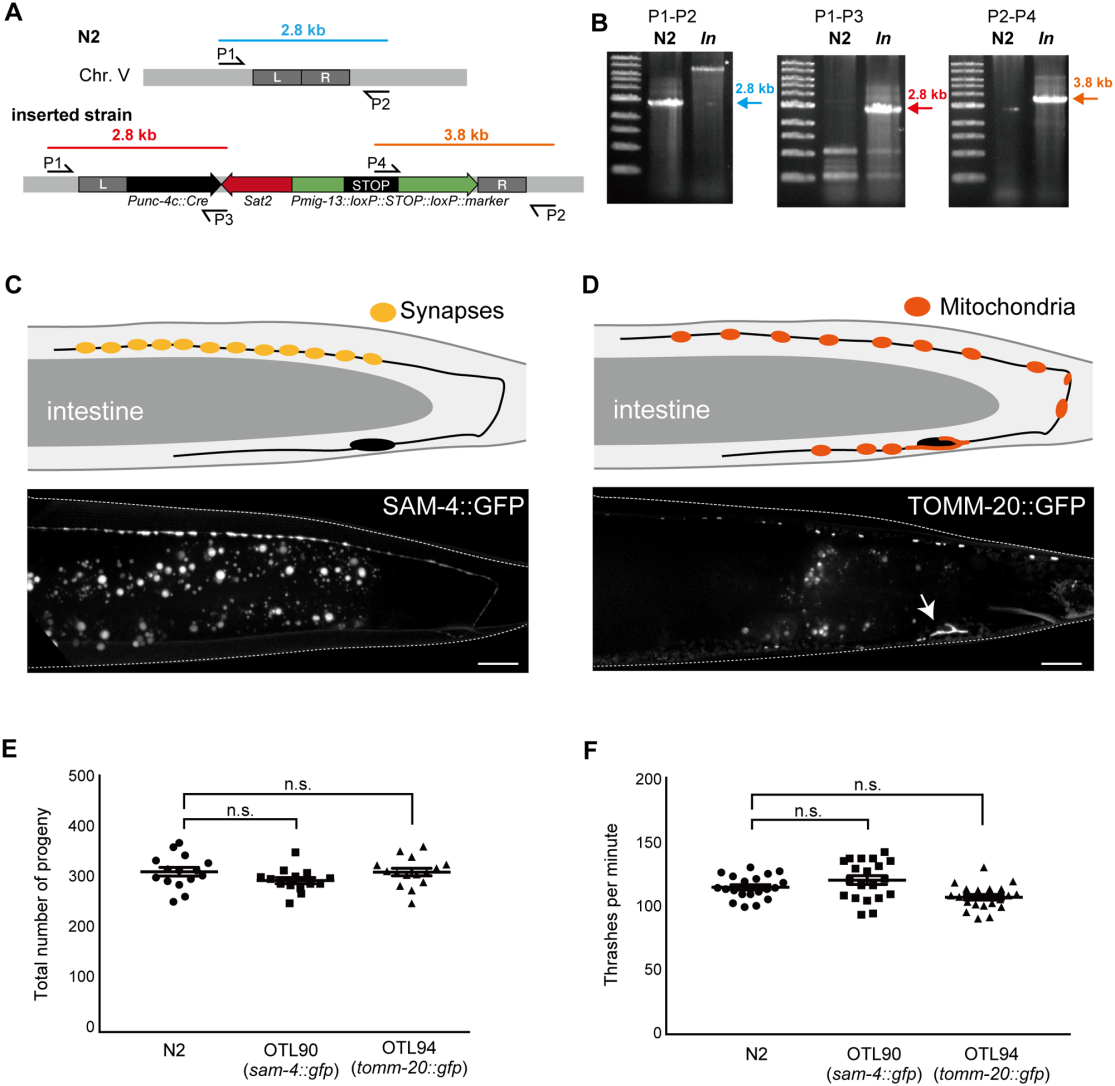
Characterization of the strains obtained by CRISPR/Cas9 and NTC selection. (A, B) Genomic analysis to confirm that single-copy insertion has occurred. (A) Scheme showing the primers used to characterize the single-copy insertion on chromosome V. In the N2 genome, the P1–P2 primers pair generates a 2.8-kb PCR product (blue). P1–P3 and P2–P4 primer pairs generate a 2.8-kb (red) and a 3.8-kb (orange) band, respectively, in the resultant strains but not in the wild-type strain. (B) Representative results from the worm PCRs. The results are consistent with the genomic analysis shown in panel A. (C) Schematic drawing of the DA9 neuron (upper panel) and a fluorescent image of the synaptic marker, SAM-4::GFP (lower panel). Note that SAM-4::GFP signals are specifically localized at synapses in the dorsal axon. (D) Schematic representation of the DA9 neuron (upper panel) and a fluorescent image of the mitochondrial marker, TOMM-20::GFP (lower panel). Note that mitochondria are localized in the cell body (arrow) and axons. Dendritic localization is relatively weak. Bars = 10 μm. (E) Total number of progeny. Statistical significance was determined by Dunnett’s test, n = 15 (N2), = 14 (OTL90) and = 15 (OTL94). n.s., statistically not significant. Compared to wild type. (F) Body bending was measured for 1 min, after 1 min adaptation of the worms in M9 buffer. Statistical significance was determined by Dunnett’s test. Twenty worms were counted for all the strains. n.s., statistically not significant. Compared to wild type.

CRISPR/Cas9-based genome engineering is performed widely in *C*. *elegans* [18, 29]. Several pairs of antibiotics and their cognate resistance genes have been used as positive selection markers in genome editing [12, 19, 33]. Here, we tested whether NTC-based selection can be reliably used for Cas9-mediated single-copy insertion. To avoid any side effects associated with insertion, we inserted the expression cassette into the well-characterized locus used for universal MosSCI [25]. A previous study exploited genomic position 8,420,204 of chromosome II (the locus for *ttTi560*) for Cas9-triggered, single-copy insertion [21]; however, we found that the transgene’s expression level was higher when inserted into chromosome V at position 8,643,066 (the locus for *oxTi365*) (data not shown). We tried to visualize the SAM-4 synaptic vesicle marker, which is fused to the green fluorescent protein (GFP) [26]. To achieve DA9-neuron-specific dominant expression, we employed a previously described strategy involving *Cre*-*loxP* [23]. The *unc-4c* promoter (*Punc-4c*) is a DA-neuron-specific promoter, whereas the *mig-13* promoter (*Pmig-13*) is a DA9- and VA12- dominant promoter. Thus, if *Pmig-13*::*loxP*::*STOP*::*loxP*::*sam-4*::*gfp* and *Punc-4c*::*Cre* are co-expressed, SAM-4::GFP should be detected only in the DA9 neuron in the tail region of the worms (Fig 3A). We attempted to insert both fragments into the same locus using CRISPR/Cas9 and NTC selection. We injected the repair template (pOB5_*sam-4)* together with the Cas9-encoding vector (pDD162), guide RNA expression vector (pTK73_pOB5) and *mCherry* vectors (pCFJ90, pCFJ104 and pGH8) (Fig 3B) into the N2 worms, after which they were allowed to lay eggs at 20°C. The next day, NTC solution was added to the plates at 0.04 mg/mL (weight of NTC/volume of NGM agar). One week after the injections, only the transgenic worms (either the single-copy inserted worms or the extrachromosomal array-positive worms) had survived on the selection plates. We isolated the L4/adult worms that showed NTC resistance but did not display mCherry expression (a marker for the extrachromosomal arrays) (Fig 3C). Note that the isolated worms are sometimes still heterozygous, therefore, we cloned out worms in the next generation to obtain homozygous worms. Integration was confirmed by worm PCR, followed by microscopic observation of SAM-4::GFP (Fig 4A and 4B). As anticipated, SAM-4::GFP was observed specifically in the DA9 neuron around the tail region (Fig 4C). These results suggest that NTC selection is useful for Cas9-mediated single copy insertion. Using the same strategy, we generated the DA9-specific mitochondrial marker as well (Fig 4D). To test whether *Sat2* expression causes some side effects in worms, we counted the total number of progeny and the bending rate of the two established lines; however, there were no statistically significant differences between N2 and each of the two strains (Fig 4E and 4F). These results suggest that *Sat2* expression has no significant influence on worm health.

### NTC and hygromycin B selection systems can be used at the same time

**Fig 5:**
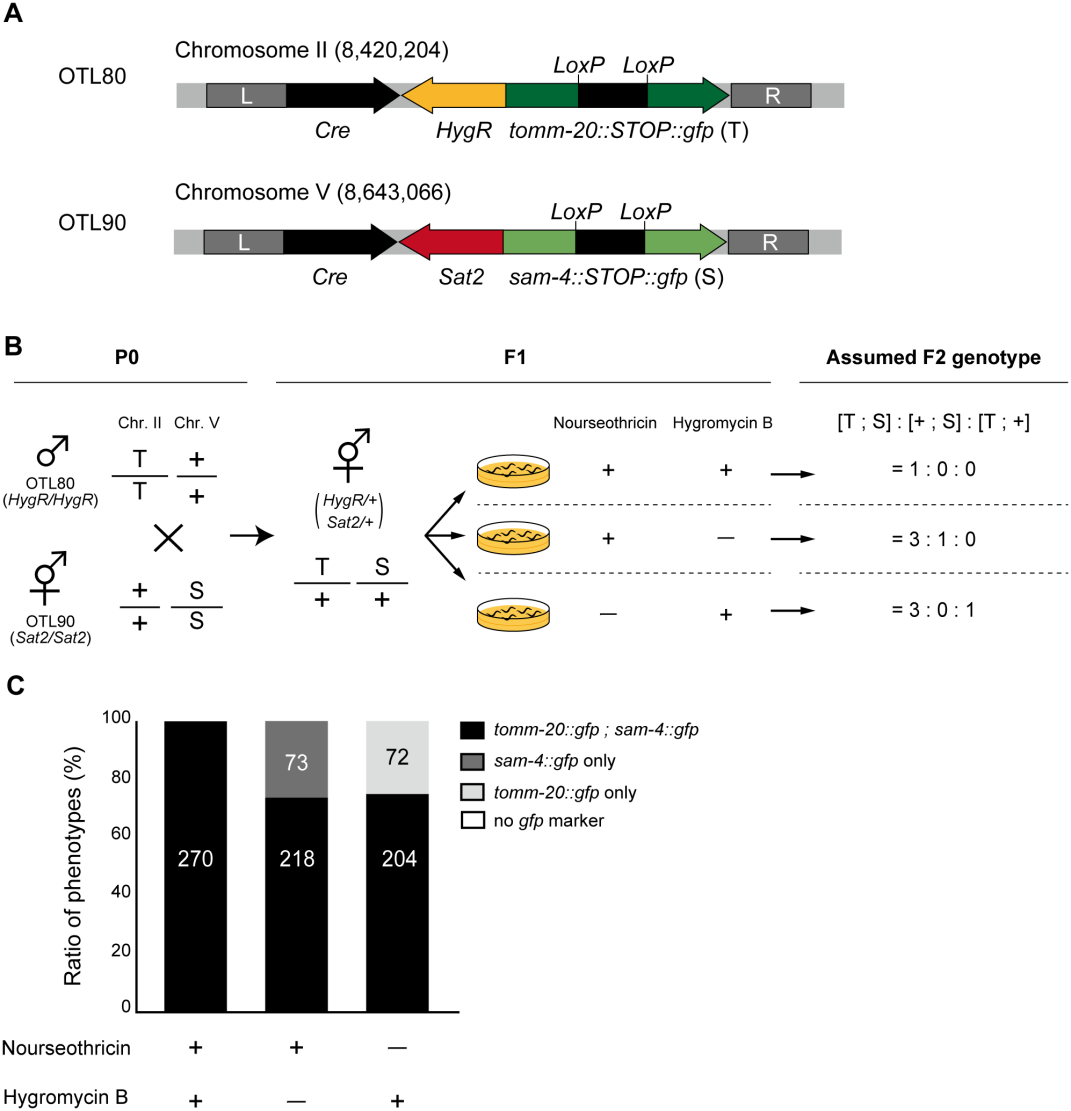
Combination screening assay for the NTC-*Sat2* with hygromycin B-*HygR*. (A) Schematic drawings showing the design of the DA9-specific expression cassette in two strains. The OTL80 strain has *HygR* and *tomm-20*::*STOP*::*gfp* inserted into chromosome II. Worms of this strain can grow on hygromycin B-containing NGM plates and they each have a TOMM-20::GFP signal in their DA9 neuron. The OTL90 strain has *Sat2* and *sam-4*::*STOP*::*gfp* inserted into chromosome V. Worms of this strain can grow on NTC-containing NGM plates and they each have a SAM-4::GFP signal in their DA9 neuron. (B) Schematic drawing showing the experimental procedure and expected result. T; TOMM-20::GFP, S; SAM-4::GFP, +: wild-type allele. After crossing, the L4 stages of the F1 generation worms were placed onto selection plates containing 0.04 mg/mL of NTC and/or 0.08 mg/mL of hygromycin B. If NTC-*Sat2* selection and hygromycin B-*HygR* selection work independently, the phenotypes of the F2 generation follow the Mendelian inheritance pattern shown. (C) Observed data: graph showing the phenotype ratios of the F2 worms. The numbers of worms counted are shown in the bars. The ratios obtained in this experiment were almost the same as those expected by Mendelian genetics.

The several antibiotic selection systems established to date include puromycin [8], geneticin (G418) [9], hygromycin B [10, 11] and blasticidin S [12]. More and more strains have been generated recently using antibiotic resistance markers, making it possible that in the near future researchers will have the opportunity to cross two strains carrying different antibiotic resistant genes. Here, we focused on widely-used hygromycin B and its resistance gene *HygR*, and tested whether our NTC-*Sat2* system can be used with the hygromycin B system. We generated the *HygR*-carrying integration strain in the same way as for *Sat2*, but the inserted marker and insertion locus was changed to TOMM-20::GFP and chromosome II respectively (Fig 5A). This strain also displays TOMM-20::GFP in a DA9-specific manner. The workflow for the combination assay is described in Fig 5B. First, we crossed male OTL80 [*HygR*, tomm-20::GFP II] worms with hermaphrodite OTL90 [*Sat2*, *sam-4*::*STOP*::*gfp* V] worms to obtain the F1 generation, which carried both *HygR* and *Sat2*. Observing the GFP signals led to easily identifiable crossed or self-fertilized worms. The L4 stages of the F1 worms carrying both *HygR* and *Sat2* were transferred onto three different types of antibiotic plates: (i) NTC only, (ii) hygromycin B only, or (iii) both NTC and hygromycin B. NTC and hygromycin B solutions were added to the plates at 0.04 mg/mL and 0.08 mg/mL (weight of antibiotic/ volume of NGM agar), respectively. When both antibiotic screening systems work well, the resulting F2 adult worms on each selection plate will carry their respective antibiotics resistance genes. We observed the GFP signals from the F2 worms as an indicator of resistance genes. Having TOMM-20::GFP (T) indicates that the worms are also carrying *HygR*, whereas having SAM-4::GFP (S) indicates they are carrying *Sat2*. If the hygromycin B selection and the NTC selection work independently, the ratio of the F2 phenotype should be as follows: [T; S]: [+; S]: [T; +] = (i) 1: 0: 0, (ii) = 3: 1: 0, (iii) = 3: 0: 1 (Fig 5B). In fact, as a result of this genetic experiment, phenotype ratios were almost the same as expected: (i) = 1: 0: 0, (ii) = 2.99: 1: 0, (iii) = 2.83: 1: 0 (Fig 5C). Worms carrying only *Sat2* were not observed on hygromycin B plates while those carrying only *HygR* were not observed on NTC plates. Only worms that have both *Sat2* and *HygR* were expanded on plates containing both Hygromycin B and NTC. This result indicates that NTC-*Sat2* selection can therefore be used with hygromycin B-*HygR* selection at the same time.

## Summary and discussion

In this study, the NTC–*Sat2* selection system was established as a dominant selection tool for *C*. *elegans* transgenesis and genome editing. NTC treatment was able to effectively prevent wild-type worm development. Notably, the wild-type worms were unable to survive in the presence of only 0.04 mg/mL of NTC, while the *Sat2*-carrying transgenic worms reproduced. *Sat2* is a small protein and did not cause defects in reproduction and movement in the worms (Fig 4E and 4F). We have succeeded in maintaining the extrachromosomal array in the strains for over three months on NTC plates without seeing any phenotypic abnormalities. However, we are unable to conclude that long term exposure to NTC will not have any detrimental effects while this is also the case in other antibiotics. Thus, for researchers wanting to use these strains in different experiments, we recommend they should be reared for at least one generation on normal NGM plates before use. For these experiments, some visible markers need to be included to pick up extrachromosomal-array carrying worms. The NTC–*Sat2* selection system is useful not only for maintaining an extrachromosomal array (Fig 2) but also for Cas9-mediated single-copy insertion (Figs 3 and 4). Heterozygous worms with a single copy *Prps-0*::*Sat2* could survive on NTC plates, indicating that even a single copy of *Sat2* can confer sufficient NTC resistance. Although the price of NTC is much more expensive ($257/g at Gold Biotechnology, St. Louis, MO) than other antibiotics such as geneticin ($28/g) and hygromycin B ($64/g), the cost of NTC selection is comparable to these widely-used antibiotics because NTC is effective at much lower concentrations (the effective concentrations are 0.04 mg/ml, 1.6 mg/ml and 0.25 mg/ml for NTC, geneticin [9] and hygromycin B [10], respectively). Since drug selection in *C*. *elegans* was established, many strains have been generated that contain drug-resistance markers. For some experiments, researchers need to generate transgenic worms in these backgrounds. As *Sat2* specifically inactivates the streptothricin-class of aminoglycoside antibiotics via its acetyltransferase activity, NTC should be used together with puromycin [8], neomycin [9], hygromycin B [10, 11] or blasticidin S selection [12, 16]. In this study, we only showed the NTC-hygromycin B pair (Fig 5), but other combinations could be also used, thereby facilitating more complex experimental designs to be undertaken in *C*. *elegans*. While this study has focused on NTC selection based on plate culture alone, the NTC–*Sat2* system will also be applicable to liquid culture protocols for expanding a large population of transgenic worms.

CRISPR/Cas9-mediated genome editing, a powerful tool in *C*. *elegans* genetics, has elucidated a broad range of biological phenomena *in vivo* [18, 19, 34]. The DA9 neuron is a good model for studying neuronal morphogenesis and axonal transport [26, 35–37]. In order to visualize organelle markers in a DA9-dominant manner, previous studies have used complex genetic systems combining single-copy insertion of *Punc-4*::*Cre* and extrachromosomal arrays that have *Pmig-13*::*loxP*::*STOP*::*loxP*, both of which require laborious work for the genetic experiments [23, 38]. In contrast, we were able to successfully insert both *Pmig-13*::*loxP*::*STOP*::*loxP*::*sam-4*::*gfp*, a synaptic vesicle marker, and *Punc-4*::*Cre* into the same locus using Cas9-mediated single-copy insertion with NTC selection. This system would make it possible to analyze other organelles by changing the markers. In fact, using the same strategy, we obtained a DA9-specific mitochondrial marker as well (Fig 4D). Moreover, monitoring cell-specific expression would be possible in other neurons using different promoter combinations.

In conclusion, as a dominant marker for gene transfer in *C*. *elegans*, the NTC–*Sat2* system offers many advantages to researchers.
